# Design, Construction and Testing of IoT Based Automated Indoor Vertical Hydroponics Farming Test-Bed in Qatar

**DOI:** 10.3390/s20195637

**Published:** 2020-10-02

**Authors:** Muhammad E. H. Chowdhury, Amith Khandakar, Saba Ahmed, Fatima Al-Khuzaei, Jalaa Hamdalla, Fahmida Haque, Mamun Bin Ibne Reaz, Ahmed Al Shafei, Nasser Al-Emadi

**Affiliations:** 1 Department of Electrical Engineering, College of Engineering, Qatar University, Doha 2713, Qatar; amitk@qu.edu.qa (A.K.); sa1401765@student.qu.edu.qa (S.A.); fa1203644@student.qu.edu.qa (F.A.-K.); jh1401965@student.qu.edu.qa (J.H.); alshafei@qu.edu.qa (A.A.S.); alemadin@qu.edu.qa (N.A.-E.); 2 Centre of Advanced Electronic and Communication Engineering, University Kebangsaan Malaysia, Bangi, Selangor 43600, Malaysia; fahmida32@yahoo.com (F.H.); mamun.reaz@gmail.com (M.B.I.R.)

**Keywords:** vertical hydroponics, indoor farming, automated system, internet of things (IoT), test-bed

## Abstract

Growing plants in the gulf region can be challenging as it is mostly desert, and the climate is dry. A few species of plants have the capability to grow in such a climate. However, those plants are not suitable as a food source. The aim of this work is to design and construct an indoor automatic vertical hydroponic system that does not depend on the outside climate. The designed system is capable to grow common type of crops that can be used as a food source inside homes without the need of large space. The design of the system was made after studying different types of vertical hydroponic systems in terms of price, power consumption and suitability to be built as an indoor automated system. A microcontroller was working as a brain of the system, which communicates with different types of sensors to control all the system parameters and to minimize the human intervention. An open internet of things (IoT) platform was used to store and display the system parameters and graphical interface for remote access. The designed system is capable of maintaining healthy growing parameters for the plants with minimal input from the user. The functionality of the overall system was confirmed by evaluating the response from individual system components and monitoring them in the IoT platform. The system was consuming 120.59 and 230.59 kWh respectively without and with air conditioning control during peak summer, which is equivalent to the system running cost of 13.26 and 25.36 Qatari Riyal (QAR) respectively. This system was circulating around 104 k gallons of nutrient solution monthly however, only 8–10 L water was consumed by the system. This system offers real-time notifications to alert the hydroponic system user when the conditions are not favorable. So, the user can monitor several parameters without using laboratory instruments, which will allow to control the entire system remotely. Moreover, the system also provides a wide range of information, which could be essential for plant researchers and provides a greater understanding of how the key parameters of hydroponic system correlate with plant growth. The proposed platform can be used both for quantitatively optimizing the setup of the indoor farming and for automating some of the most labor-intensive maintenance activities. Moreover, such a monitoring system can also potentially be used for high-level decision making, once enough data will be collected. This work presents significant opportunities for the people who live in the gulf region to produce food as per their requirements.

## 1. Introduction

The world population is increasing every day and it is expected to reach 9.3 billion in 2050 [1]. Therefore, crop production has to be increased in order to maintain a sufficient amount of food. However, the production of crops is affected by many factors like the unusual weather changing, lack of water and the lack of sufficient arable lands available to grow the crops [1]. As a result, people started to use different methods of farming to reduce water consumption and the space for farming, one of the most famous methods is the vertical hydroponic farming. Vertical hydroponic farming is a combination of two old methods, which are vertical method and hydroponic method. These methods are old, but recent research and studies by scientists worldwide have proved its usefulness [2,3,4]. The hydroponic system is a method that depends on growing the plants in the water without the use of soil, it has been proved that the plants do not need soil as long as the essential nutrients, minerals and the suitable pH maintained stable within a certain range inside the water [1]. There are different types of hydroponic systems that are known, such as wick system, drip system, nutrient film technique (NFT), deep flow technique, and aeroponic system [1]. The hydroponic systems are currently developed to solve the problems that affect the plant growth by controlling all the parameters automatically, which made it possible to make an indoor farming without consuming large space of land [2]. The automatic vertical hydroponic systems portend a huge revolution in food production, where different kinds of crops can be grown in homes that can satisfy peoples’ needs.

**Figure 1:**
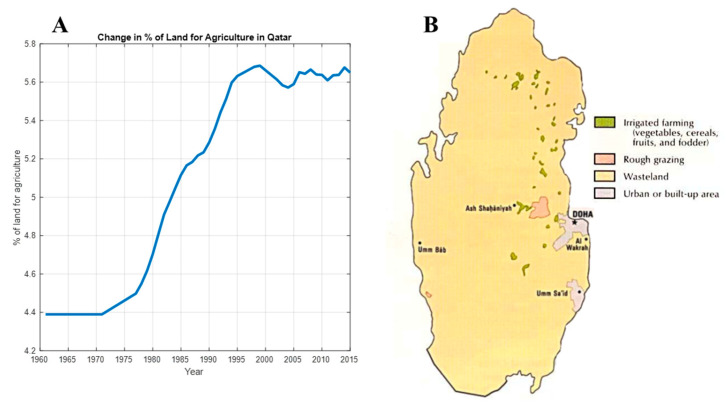
(**A**) Change in % of land for agriculture in Qatar; (**B**) distribution of agricultural land in Qatar.

Gulf countries have experienced large demographic growth in the recent years. This considerable growth requires a local increase in agricultural productivity to maintain a sufficient amount of food that satisfies the population needs. Generally, agriculture is a strategic sector in such countries since it has a crucial role in food security and reduces dependency on imports. Clearly, such countries depend on imports to meet 90% of its food and water consumption needs [5]. Importantly, Qatar depends on imports due to its limited arable lands, low rainfall, dry and hot climate [6,7,8,9], scarcity of groundwater and high evaporation rates [10]. Indeed, there has been slight increase in the percentage of arable land over the years, Figure 1A, and currently 6% of the total Qatar territory is arable, which is about 650 km^2^ or 65,000 hectares in Figure 1B [11]. The local market needs 2, 260,000 t of vegetables in a year, whereas the agricultural land is a very small share of the total land, being 6% of the total land, as shown in Figure 1 [12,13].

The Qatar National Food Security Program (QNFSP) was launched to ensure and promote food security and self-reliance in food production by using technology and modernized techniques in agriculture, which will ensure high crop production all year-round, conservation and optimal use of the natural resources. After the blockade, strong initiatives were taken by the government to tackle the local food demand and as well as having complete self-sufficiency. One of these initiatives with government support, helped locally-owned company Baladna cover 95% of the country’s dairy products, as well as exporting to some countries [11]. Similar efforts were made in the agriculture and aquaculture market. Prior to the blockade, Qatar imported 85% of its vegetables; this program plans to increase self-sufficiency from 10% to 60% by 2023 [5]. Therefore, a modernized and indoor agricultural system should be developed to help in achieving the aims of Qatar National Food Security Program. This paper is expected to raise awareness about the importance of hydroponic system in food self-sufficiency and encourage them towards sustainable agriculture sector in gulf countries showing Qatar’s perspective as a case study. In addition, it is expected to guarantee the effective participation of locals in term of cultivating their food needs in their homes using small space or room without the need of having greenhouse-based farms or investing millions of dollars to make suitable agricultural land.

Several studies on the design, implementation and evaluation of the hydroponic systems were reported in the literature and there are some comprehensive review articles summarizes the recent developments [14,15,16]. Rius-Ruiz et al. (2013) [17] made a computer-based vertical hydroponic system that analyzes and determines the optimal nutrient solution for tomato plant with low cost. While Ibayashi et al. (2016) [18] made a reliable wireless controlled hydroponic system for tomato plants. The system was focusing on making a reliable wireless sensor network (WSN) using different frequency bands based on IEEE standards. However, both systems neither monitor all the necessary parameters nor can control them automatically. Montoya et al. (2017) [19] built an automated aeroponic system that is based on Arduino platform to measure the important system parameters such as temperature, humidity, and potential of hydrogen (pH) levels. However, the system is highly expensive in comparison with the other hydroponic systems. Eridani et al. (2017) [20] made an automatic NFT system, which was based on a prototype scale with a microcontroller, total dissolved solids (TDS) sensor, and proximity sensor. The system can detect the electrical conductivity and deliver the water automatically in the case of water level decrease. However, the system did not use pH sensor which is crucial for any hydroponic system. Ruengittinun et al. (2017) [21] developed a controllable hydroponics system using Internet of Things (IoT), microcontrollers and sensors to observe and control the temperature, electrical conductivity, humidity, and pH using an Android application. However, the system depends on user intervention through the mobile application. Palande et al. (2018) [22] have designed an automated system for indoor hydroponic system using microcontrollers, sensors, and Internet of Things (IoT). The system is capable to control and monitor all the required parameters to have healthy indoor plants growth such as temperature, lights, humidity etc. But the planting structure is not suitable for plantation rather it is a prototype to exhibit sensing and control system. Sirawattanakul et al. (2018) [23] implemented a smart vertical farm system using light controller, temperature and humidity sensors, nutrition mixer, and the IoT platform. IoT platform was used to monitor and control different parameters which are important in plant growth via the internet. However, this study did not include system design and evaluation steps which could be useful for other researchers to reuse the concept. Tagle et al. [24] have demonstrated an automated monitoring urban farming system which was built-in from scratch. The indoor hydroponic tower-based system can measure the system parameters. However, it was not interfaced to any IoT platform for remote monitoring. Cambra et al. [25] proposed an auto-calibrated pH sensor which can detect and adjust the imbalances in the pH levels of the nutrient solution used in hydroponic system to control the irrigation for the plants. Although the system is designed with control and automation, however, it is not a full-featured hydroponic system with IoT platform. Marques et al. [26] proposed an iHydroIoT platform for data collection and an iOS mobile application for data consulting and real-time analytics. This system is not only displaying temporal changes monitoring of light, temperature, humidity, CO_2_, pH, and electrical conductivity but also water level for enhanced hydroponic supervision solutions. However, this system was not tested for plant growing structure rather it was evaluated for electronic monitoring and control. Ruscio et al. [27] reported a low-cost vertical hydroponic system using Zipfarm towers and sensor to measure temperature, water level, humidity, pressure, light intensity, pH and electric conductivity without requiring any human intervention. However, the systematic design and calibration were not reported in their work and the IoT platform was not deployed in that work as well. Van et al. [28] an IoT-based intelligent hydroponic system call PlantTalk, which can perform automatic LED lighting, water spray, water pump control etc. through the smart phone application. However, design steps and application are open-source and the system is not suitable for growing enough plants for a household requirement.

Different machine learning (ML) algorithms such as Bayesian Network [29,30], neural networks [31], fuzzy logic [32,33], k-nearest neighbors (kNN) [34] and deep neural network [4,29] to appropriately control the hydroponic environment based on the multiple input parameters gathered to enhance the performance of the automated hydroponic systems. The new direction of the works proposed in these ML related articles are interesting and demand further investigation with our proposed system once a large amount data is collected through the IoT system. Recently a study has been reported the promising prospect of aquaponics for the sustainable food security solution for Qatar [35]. However, there is no technical details of such a solution was provided in the work to achieve a sustainable food security solution which was a major motivation for this study.

Various vertical hydroponic systems such as A-frame, Zig-zag, vertical hydroponic tower, ZipGrow tower and vertical Nutrient Film Technique (NFT) system were available. Amongst the various vertical hydroponic systems available, the concept and design of the vertical NFT system was considered to be used for this study [15,36,37,38]. It is due to the simplicity in the design, ease in its assembly, easy configurability of the LED, high productivity of plants within a small place, and the strong supporting system that holds the structure. Many organization, such as Plenty, Bowery, Aerofarms, and Ikea etc., are commercially producing different vegetables in urban areas using proprietary architecture and technologies with the help of the professionals using large scale indoor farming with high scale investments [39,40,41]. Even though these are helping fulfilling food demand and promoting urban farming, however, these are not suitable for individual level and cannot be done by the people without technological knowledge.

Our aim is to develop a simple, automated, and scalable cost-effective system which can be used by general people from the gulf countries like Qatar for personal farming as well as large scale farming. External environmental conditions in gulf countries during the summer are not in favour of farming and greenhouse and indoor farming with continuous air conditioning is a must for farming. However, every house can be a small farm utilizing a small space of the house/flat without much increasing water and power consumption while can grow the basic needs of the households. This is the major motivation of this project and Qatar government has declared subsidization for such effort.

All the outlined works were focusing different aspects however none of them was designed and implemented to promote indoor farming solution for a deserted country where it is not possible to carryout farming throughout the year without expensive greenhouse-based structure. While some works were emphasizing automation and monitoring and others were showing the importance of IoT, none of them was a complete solution with monitoring and controlling the various hydroponics elements for indoor application with a reasonable structure to produce required vegetation for a small family. They missed a very important aspect of monitoring power consumption along with temperature and humidity control, which is crucial for a desert state such as Qatar. The studies used intelligent algorithms that showed better results in optimizing controlling parameters, but they have not considered the power consumption and cost effectiveness for an automated indoor farming system in a personal space. Moreover, the studies did not show the detailed design steps involved in developing such an automated system. The machine learning used in the previous studies cannot be generalized as they are for specific vegetation, whereas the study presented in the paper can take advantage of the ML based systems to have better control mechanisms for different vegetation than the one used in the study. Therefore, this study represents a detail step by step description of the design and implementation of a cost effective vertical automated IoT based indoor hydroponic monitoring and controlling system.

The major contributions of the work are: (1) detailed steps in designing and constructing an indoor automatic vertical hydroponic system, (2) calibration procedure for various sensors needed in such a system, (3) complete IoT based solution that can be implemented in any personal space, and (4) developed framework for further research for a sustainable solution that does not depend on the outside non-favorable gulf climate. As per our knowledge, this is the first full-scale implemented vertical hydroponics system with the system components elaborately mentioned along with cost and power consumption analysis while considering in-house farming in the GULF region. The cost-effectiveness of our system makes it suitable for personal farming. Our IoT based automatic system observes and maintains the environment of the hydroponic system in real time and provides user the facility to monitor remotely using web interface using computer/mobile and a mobile application and update immediately if the user intervention is required. The rest of the paper is arranged as follows: Section 2 discusses the design steps of the implemented hydroponic system with details of the sensors, IoT platform, and other system components. Section 3 provides the details of how each system component was calibrated and tested. Section 4 provides the results of the implemented system while Section 5 discusses the complete system and compares it with some other commercial and research solutions followed by a conclusion and future directions of research in Section 6.

## 2. Materials and Methods

**Figure 2:**
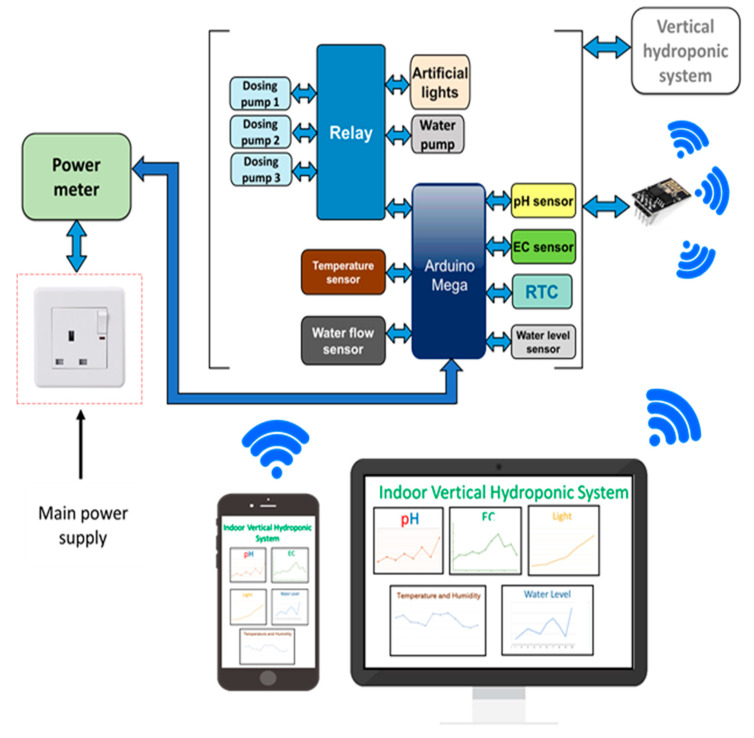
Block diagram of the designed system.

The block diagram of the automated vertical hydroponic system consists of six parts: main power supply, power meter, sensing and control system, vertical hydroponic structure, Wi-Fi module, and online database. The overall block diagram is shown in Figure 2. All the sensors connected to the vertical hydroponic system can be monitored from the IoT platform on any smart device. There is a power meter module for continuous monitoring the power consumption of the system in order to make the system power efficient and possible for large scale expansion. Each part of the block diagram will be discussed in detail in the later sections of the paper.

In any hydroponic system, there are several parameters that should be maintained within certain range, such as pH, electric conductivity (EC), temperature of the surroundings, and water level of the container. An automatic hydroponic system should adjust and maintain these parameters within its suitable value automatically and independently without the requirement of user intervention. Different sensors are connected to the microcontroller to monitor the different parameters of the hydroponic system. A panel electromechanical relay was used to control artificial lights, water pumps and the dosing pumps that were used to add pH and nutrient to the water. Finally, all the acquired data from the central microcontroller circuit was sent wirelessly to Thingspeak (Dublin, Ireland) [42], online database through an ESP 8266 Wi-Fi module (Shanghai, China) [43], the details of the sensors and materials used in the paper can be found in the Supplementary Table S1. The design of the whole system could be divided into the below subsections:

1. NFT structure and essential components
2. Water flow path
3. Nutrition and pH controlling system
4. Internet of Things (IoT) platform

### 2.1. NFT Structure and Essential Components

**Figure 3:**
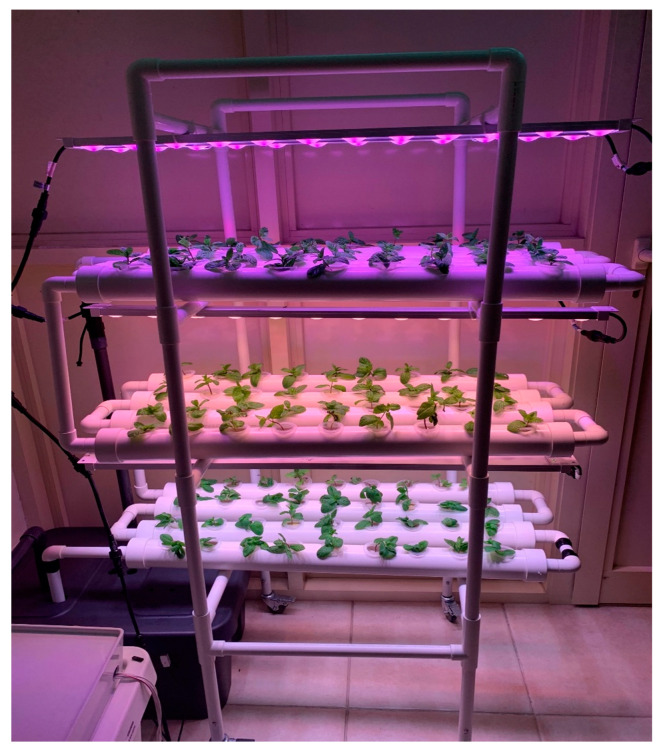
Vertical NFT hydroponic structure assembled and connected to the monitoring and control system.

The hydroponic system was chosen to be a vertical NFT hydroponic system since it has the greatest benefits compared to the other systems [44]. For this paper, the vertical NFT system made by Koray Company (Guangdong, China) [45] was selected, which is consisting of 3 shelves. Each shelf is holding 4 polyvinyl chloride (PVC) pipes with a diameter of 63 mm (0.063 m), and each pipe consists of 9 planting holes with a diameter of 32 mm (0.032 m). The dimensions of the vertical NFT hydroponic systems are 96 cm (L) × 50 cm (W) × 90 cm (H) (Figure 3). The PVC system was purchased off-the-shelf to reduce development time. However, given the known specification it can be manufactured and assembled locally, which will reduce the cost significantly.

A typical hydroponic system consists of the hydroponic pipes, nutrient container, water pump, artificial lights, nutrient, and pH adjustments’ solutions. The nutrient container, water pump, and artificial lights is selected carefully to assure the highest efficiency of the hydroponic system. This section presents the process of selecting the right size of the nutrient container and the water pumps, the selection of the artificial lights, nutrients, and pH adjustment solutions.

#### 2.1.1. Nutrients’ Container

A nutrient container is used to reserve the nutrient solution that would be supplied to the vertical NFT system. Since it is a closed system, all the excess solution would return to it. The ideal container should be made from plastic material, using metal containers is prohibited as they are reactive materials. Moreover, it should block the light to pass through it to prevent the formation of algae [46]. The size of the container should be big enough to hold the right amount of the solution, and this amount is decided according to the number of the plants in the system. Number of the plants between 40–50 should use a minimum of 5 gallons (18.9271 L), by adding another 20–25 plants, the amount would be increased by 1 gallon (3.78541 L) [46].

The vertical NFT system has 4 pipes distributed in 3 shelves and each pipe has 9 holes to grow the plants, and the number of plants in the system can be calculated:

*Number of plants* = *number of pipes* × *number of shelves* × *number of plants holes* (1)

So, the *number of the plants* can be grown in this system is 4 × 3 × 9 = 108. The plants were divided into 4 group of: 50 plants, 25 plants, 25 plants, 8 plants. The first group of 50 plants would use 5 gallons of water (18.9271 L), the second and the third group of the 25 plants would use extra 2 gallons (7.57082 L), for the last 8 plants, it would use 8/25 gallon of water (1.21133 L), the overall size of the container would be 7.32 gallon (27.70921 L). The amount of nutrient solution needed to be inside the container was almost equal to 28 L. However, since the NFT system would have 30% losses of water due to the consumption of plants and water evaporation from heat, 30% of 28 L is equal to 8.4 L, for that an extra 9 L was added to compensate any water losses, which gave in total 37 L inside the container. For this design, the container size was equal to 46 L in order to leave some space on the top of the container for the exposure to air.

The inner dimensions of the container were 35 cm (L) × 47 cm (W) × 28 cm (H), which would give a volume almost equal to 46 L. The container was filed with a 37 L of water and the water needed to reach a certain height depending on the container dimension, which was calculated using Equation (2).

(2) $$Water\;head\;height=\frac{Liter\;of\;water\times 1000}{L\times W}$$

The *height of water* $=\frac{37\times 1000{\;\mathrm{cm}}^{3}}{35\;\mathrm{cm}\times 47\;\mathrm{cm}}=22.4\;\mathrm{cm}$. Therefore, for 37 L of water, the equivalent height of the water inside the container will be almost equal to 22.5 cm.

#### 2.1.2. Water Pump

Sizing the water pump is very important as selecting the right pump would assure the sufficient amount of water is flowing continuously in the system. For this design, submersible pumps were selected to be used since they suit the small systems that needs 1200 of gallons or less. The sizing of the pump was done in three steps. The first step was to calculate the gallons per hour (GPH) that the pump was required to supply to the system, and the second step was to measure the head height of the hydroponic system and the third step was to use the first two information to verify if the pump was suitable using the water pump datasheet [47].

The calculation for the GPH of water that the pump needs to supply to the system every hour can be found in the Supplementary Table S2. It is important to consider the loss in efficiency between 15–30% of the pump in the calculations. According to Supplementary Table S2, the NFT system requires 4–6 GPH per trough, by selecting the average value of the *flow rate* as 5, and the worst-case scenario in the *loss in efficiency* as 30%, the *total GPH* was calculated using Equation (3).

(3) Total GPH=number of pipes×flow rate GPH×loss in efficiency

$$\mathrm{Total}\;\mathrm{GPH}=12\times 5+(12\times 5\times \frac{30}{100})=120.3\;\mathrm{GPH}\;(455.4\;\mathrm{Litre}\;\mathrm{per}\;\mathrm{hour}\;(\mathrm{LPH}))$$

The head height distance was found by calculating the distance between the surface of the water and the entering point of the nutrient water to the NFT vertical system which is equal to 66.5 cm (0.665 m). The right size of the pump was selected according to the total GPH and head height distance. The pump should be able to supply at least 456 LPH water and it should be strong enough to push the water up to a height of 0.665 m or more. For this study, SUNSUN Submersible Water Pump (Chennai, India) [48] was selected, which fully satisfied the needs of the designed system. It can pump 600 LPH, and can pump up the water to maximum height of 1.3 m. Moreover, this pump does not make any noises while it is running which is important for indoor system [48].

#### 2.1.3. Artificial Lights

Lighting is essential for the plants as it plays an important role in the photosynthesis process. Any lack of lights would limit the photosynthesis which affects the growth of the plants [49]. Plants do not require the full range of light spectrum for growth. They only absorb their needed amount of light in the spectrum. The lights requirement is between 400 and 700 nm which lies within the visible range and it is also known as photosynthetically active radiation (PAR). Moreover, a high intensity of red and blue lights is needed to grow flowering plants, while for non-flowering plants need a high intensity of red light only [50].

In this design, the LED lights from Koray Company (Guangdong, China) [45], 3 lights for three tiers of the NFT system were used. Two different types of LED lights: 6K3R4 and K6 (Guangdong, China) [45] were used in this study. The specification of each set of the lights can be found in the Supplementary Table S3. The light spectrum (Supplementary Figure S1) shows that 6K3R4 has high intensity of the red light, which made it suitable for growing leafy vegetables while K6 has high intensity of blue and red lights, which made it suitable for growing flowering plants. Typically, the LEDs should be turned on for 16 h a day [51] and therefore, the lighting was controlled using a relay from the central microcontroller.

#### 2.1.4. Nutrients and pH

There are 17 different type of elements that are important for the complete growth of the plants. In the hydroponic system, these 17 elements were added to the plants as nutrient solution. However, the amount of nutrition must be added within the suitable range for each plant where any incorrect amount added can cause a huge damage to the plants. To confirm the correct range of nutrient amount in the solution, the electrical conductivity (EC) was measured continuously. Furthermore, the amount of pH must be monitored in the nutrient solution as it is considered essential for plants’ growth. Atlas Scientific pH Kit 0–14 pH sensor (Long Island City, NY, USA) [52] and Atlas Scientific Conductivity Kit K 1.0–5200,000 μS/cm sensor (Long Island City, NY, USA) [53] were used for real-time pH and EC measurement. The suitable pH range in the hydroponic system is between 5.0 and 7.5. A higher amount of pH in the nutrient solution from the recommended range for the plants leads to a higher chance of nutrient deficiencies, which become toxic to the plants. The suitable EC and pH ranges are presented in the Supplementary Table S4 for different plants. For growing plants from stem, it is recommended to lower the EC range until the plant develop a good number of roots [54].

The nutrient solution used for the plants was produced by Fox Farm (Samoa, CA, USA) [55,56]. All 17 elements come in one container and it contains three percent of nitrogen, two percent phosphorus and six percent of potassium, which are the main elements for the plants’ growth [55]. The pH up and down solutions from General Hydroponics Company (Santa Rosa, CA, USA) were used to adjust pH concentration in the nutrient solution [56].

#### 2.1.5. Isolation Circuit

The isolation circuit is an important part of the automated vertical hydroponic system. It is used to prevent any unwanted voltage and current interference that can affect the sensor readings. This unwanted current arises from ground loops and 50 Hz AC supply of the water pumps, which can affect the reliability of the sensors’ readings as these sensors are very sensitive. An inline voltage isolator chip (Atlas Scientific Basic EZO™ (Long Island City, NY, USA)) was used for galvanic isolation and improved noise immunity for power and data lines of pH sensor [57]. It is recommended to use the isolation circuit for either pH or EC sensor.

#### 2.1.6. RTC Circuit

The DS3231 real time clock (RTC) (Arduino, CA, USA) was used to calculate the real time for the system so that the events of decision making (light on/off) can be done according to real clock [58]. The module was able to retain the timing information even if there was an interruption in the main power as it is equipped with a coin cell battery.

#### 2.1.7. Air Conditioner (AC) Controlling Subsystem

**Figure 4:**
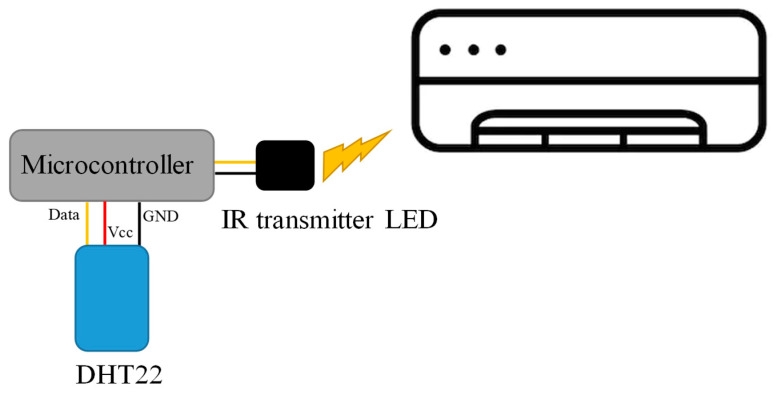
Block diagram of AC controlling subsystem.

It is essential to maintain the temperature in a specific range for the healthy growth of plants in an indoor hydroponic system. Plants require a certain range of temperature, which allows the suitable environments for the plants to grow healthier, and better. The optimal range for temperature is between 19–28 °C [59,60]. In order to control the temperature and the humidity of the room, an air conditioning subsystem (Figure 4) with automatic triggers to turn the AC on or off was implemented [61]. The system used the data coming from the temperature sensor, and through the infrared (IR) transmitter LED, it can send a signal to the AC to control [61,62].

#### 2.1.8. Power Consumption Monitoring Subsystem

The amount of power consumed has to be monitored and controlled to reduce the overall running costs of the system. The power, voltage and current consumed by the system were measured by using the power meter designed and implemented.

**Figure 5:**
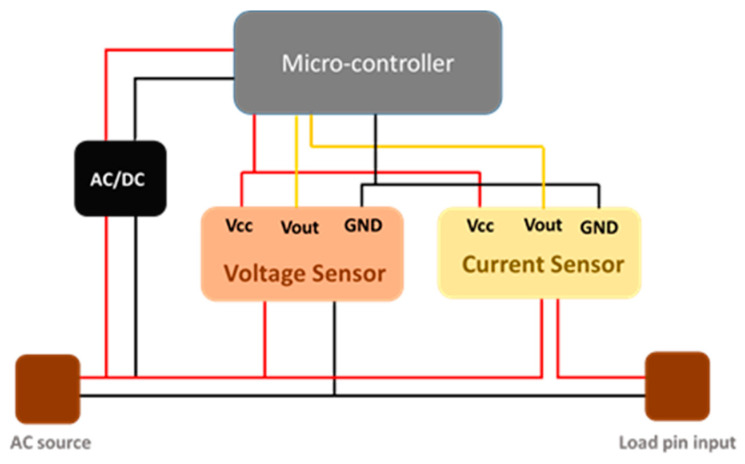
The block diagram of the power meter.

The power meter has two ports: input and output, where the input of the power meter is connected to 240 V alternative current (AC) source and the output port is connected to the hydroponic system (load). The power meter consists of micro-controller, AC/DC converter (HLK-PM12 (ShenZhen, China)) to power-up the micro-controller (Arduino Nano (Somerville, MA, USA)), a current sensor (ACS712 (Worcester, MA, USA)) and a voltage sensor (ZMPT101B (Guangzhou, China)) to measure the current and the voltage consumption of the loads, details are provided in the Supplementary Table S1. All the measured data were sent to the central micro-controller to measure the total power consumption (Figure 5).

The power consumption of air conditioning unit was monitored using a commercial power meter which was digitized to store data for evaluation. The cooling capacity of 2 ton is equal to 7.034 kW of power and power consumption of AC is cooling capacity/Energy Efficiency Ratio (EER). A two-ton air conditioner typically consumes 2.6 kW while EER = 2.7 for a typical 3-star AC. AC consists of two units, Indoor unit which is called the evaporator and the Outdoor unit which is called the Compressor. The power consumed by the evaporator is very less as compared to the compressor. Compressor unit starts only when the indoor temperature is more than desired temperature and stops once the desired temperature is achieved. Assuming 60% of the total on time compressor unit is on, energy consumption per hour of running is 1.56 kWh. However, overall monthly consumption depends on the daily usage. In Qatar, from March (18–27 °C) till May (26–39 °C) and September (28–39 °C) till November (20–30 °C) (please refer to [63,64] for more details of temperature profile), the air conditioner is typically remains ON for few hours in a day and it typically runs for longer duration during May to September [65]. Since the hydroponic system was placed in a room, where air conditioner is typically maintained by the room-users however, it was not known to us the difference between the regular usages in comparison to the usage due to hydroponic system. Therefore, a typical monthly usage of the air conditioner was logged, and the usage of the same air conditioner was logged when it was controlled by the hydroponic system to investigate the difference in monthly consumption. It is important to see whether there is a major difference in the power consumption due to hydroponic system or not.

### 2.2. Water Flow Path

The complete water flow path was illustrated using the Supplementary Figure S2. It was made using the containers, water pumps, pH, EC, water level, water flow, and temperature sensors.

### 2.3. Nutrition and pH Controlling System

**Figure 6:**
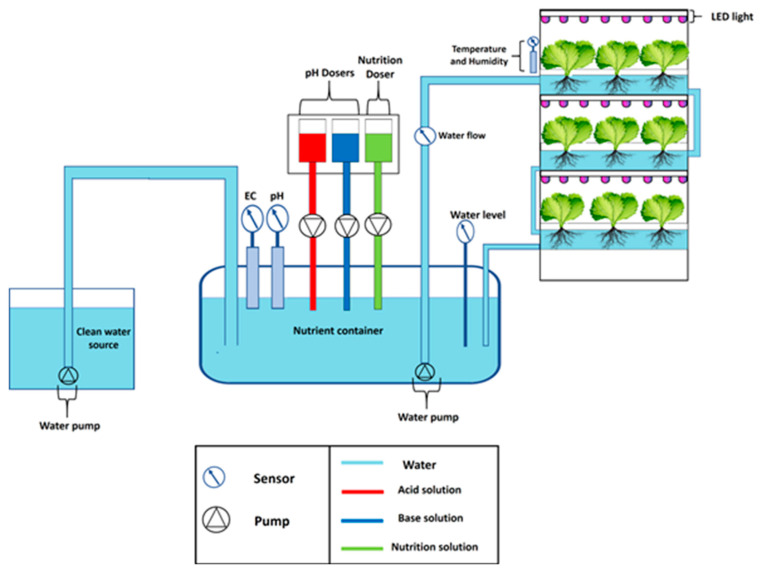
Schematic diagram of the complete system.

In order to house all the dosing pumps, a custom box is designed. From the back of the box, there are 3 holes that can allow the electric wires of the dosing pumps to be connected to the relay and microcontroller. The nutrient and pH controlling system along with the holding box for the dosing pumps can be seen in Supplementary Figure S3. The overall system schematic can be seen in Figure 6.

### 2.4. Internet of Things (IoT) Platform

Internet of things (IoT) allow the system to be connected to internet to gather and store all the information’s or data collected from the devices through a web server. The user would have the ability to reach this data through a computer or smart phones from any place and any time via the Internet [43].

In this paper all the reading of the sensors such as: pH, EC, water level, humidity, and temperatures sensors along with the power consumptions of the system were sent to web server, through a Wi-Fi-module, ESP8266 (Shanghai, China) [43]. The Wi-Fi-module is connected to the microcontroller, where all the data from the sensors were acquired and were sent to an open source IoT platform, Thingspeak [42]. Thingspeak is not only open source, but it also allows MATLAB (The MathWorks, Inc., Natick, MA, USA) based data visualization and algorithm implementation on the acquired data, which has made it as an excellent choice for indoor vertical hydroponic system. Classical and deep machine learning algorithms can be easily implemented on this platform, which is the future direction of this work. Moreover, Thingspeak supports 8 different fields, where 8 different system parameters can be displayed real-time without paying any subscription fee and the channel is updated every 20 s in the free version which is reasonably suitable for this application. Moreover, there are several applications available in the android platform which can be configured easily to access the channel in the mobile application without requiring sign-in every time in the web-browser to visualize the system parameters. Therefore, to make a cost-effective system with reliable IoT platform facilities Thingspeak based system is a suitable option. Moreover, it allows to export historical data in CSV format for further analysis, which will help significantly for future machine learning based studies.

## 3. Testing and Validation

**Figure 7:**
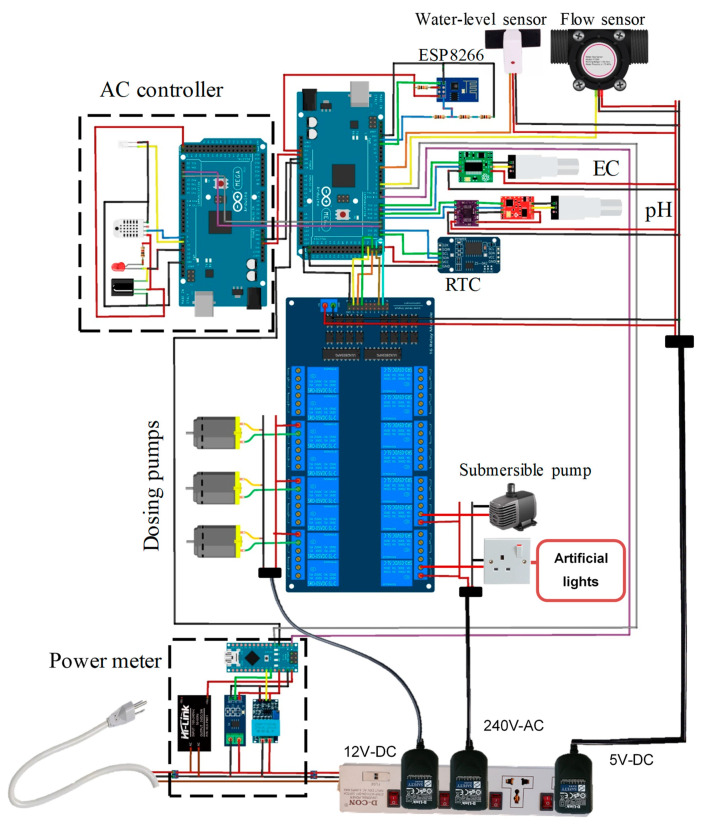
Overall circuit diagram of the system.

**Figure 8:**
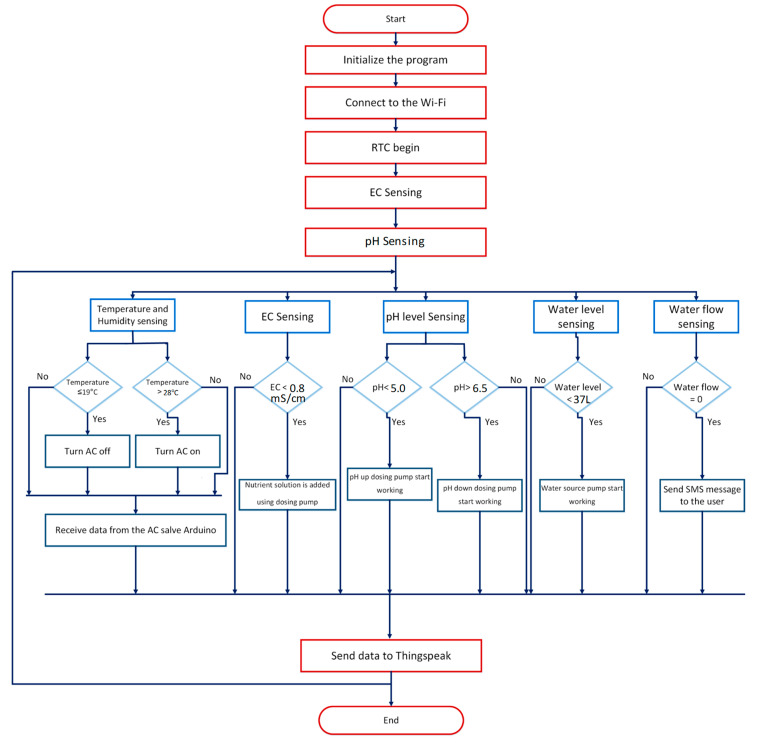
Flowchart of the automatic hydroponic system.

Various sensors used in the design had to be calibrated before using them. The overall circuit diagram of the system is shown in Figure 7, which represent all the sensors and the AC appliances connected with each other along the connection of the AC controller and power meter subsystem. Two different types of Arduinos were used, which are Arduino Mega (Somerville, MA, USA) and Arduino Nano (Somerville, MA, USA), details in supplementary Table S1. The AC controller is designed around Arduino Mega and the power meter using an Arduino Nano and both of them send data to the master Arduino Mega through serial communication. Figure 8 illustrates the working principle of the complete automatic hydroponic system based on the sensor reading.

### 3.1. Testing of Electronic Sensors

#### 3.1.1. EC and pH Sensor

The EC and pH sensors were tested and calibrated by connecting it to the Arduino Mega (Somerville, MA, USA) microcontroller. The process of calibration was done by following the instruction in the datasheet of the manufacturer. The EC sensor was calibrated using dry, low, and high EC calibration using solutions with different EC level. It was noticed that the sensors produce very accurate results (Figure S4). The pH sensor was calibrated using solutions with different pH level and results were very accurate. EC and pH are important factors to sustain healthy growth of the plants. In the system implementation, EC was added according to the instruction in the nutrient solution container, where every gallon needs 29.75 mL of nutrient solution. In this system, the required amount of water was 37 L, which is equivalent to 3.21 gallons. Therefore, to find the required amount of *nutrients*, the following equation was used:

(4) $$Nutrient=\frac{3.21\;\mathrm{gallon}\times 29.57\;\mathrm{mL}}{1\;\mathrm{gallon}}=94.9\;\mathrm{mL}\approx 95\;\mathrm{mL}$$

Therefore, initially 95 mL *nutrient* was added to the nutrient container. The EC sensor was then continuously monitoring the EC level to maintain the EC in the required level by adding water to decrease EC or adding nutrient by the dosing pump to deliver nutrient to keep it in the desired level. Furthermore, the pH is controlled by two dosing pumps where pH up or down was added to the nutrient solution to keep the pH in the suitable range for the growing plant.

#### 3.1.2. Water Flow Sensors

The YF-S201 Hall-Effect water flow sensor (Ahmedabad, India) (supplementary Table S1) was connected to microcontroller, as shown in the Figure 7 and it was calibrated to measure the flow rate. The water flow sensor was capable of measuring accurately the flow rate utilizing the hall sensor to deliver pulses on water fall and microcontroller was used to count the number of pulses to measure the flow of water. Following mathematical equations were used to calculate *flow rate* and *total volume of water supply*:

*Sensor Frequency* (Hz) = 7.5 * *Q* (L/min), where *Q* is flow rate in L/min

*Flow Rate* (L/h) = (*Sensor frequency* × 60 min)/7.5 (5)

*Litres* = *Q* × *time elapsed* (s)/60 (s/min) (6)

*Litres* = (*Frequency* (Pulses/s)/7.5) × *time elapsed* (s)/60 (7)

*Litres* = *Pulses*/(7.5 × 60) (8)

It was important to ensure the correct volume of water to add in the system when necessary and also to measure the circulation of *total volume* of water in the system. This helps us to measure the volume circulation per day and on average in a month how much fresh water was refilled to maintain the water level correct in the system.

#### 3.1.3. Water Level Sensor

The non-contact capacitive water level sensor was continuously reading ‘0’ when it senses there is liquid at the sensor level (22.5 cm height or 37 L of nutrition). However, if the water evaporates or loses due to the consumption of the plants, the water level sensor outputs ‘1’ which triggers ON the fresh water pump until it delivers 2 L of fresh water from the reservoir so that pump does not need to be turned ON frequently. In the testing phase of the system, this operation principle was tested and confirmed its functionality.

### 3.2. Dosing Pumps

In the calibration stage, it was tested that how much solution the dosing pump can deliver per minute. This was done by pumping water from a bottle to another mL labelled bottle by continuously running the dosing pump for one minute. It was observed that the dosing pump can deliver 95 mL of fluid in one minute and 1.58 mL/s. The dosing pumps were not turned ON initially during system initialization rather when the system was running for a while (after first 30 min), the EC and pH level were evaluated and based on that 5 mL of EC and pH up/down were injected in the system in each injection, however after each injection, the system waited for 10 min before next injection as it takes time to stabilize the EC and pH of the system. During this period, the dosing pumps were not activated based on the level of EC and pH. Rather, these quantities were monitored only.

### 3.3. Power Meter Subsystem

During the calibration and testing phase, the power meter subsystem was used to test power consumption of each individual system component so that overall system power consumption can be calculated and verified with the power data provided in system run-time. Table 1 shows the power consumption measured by the meter based on their daily operating time. However, the air conditioner which is the most power consuming system was investigated in two different conditions: (1) in a typical operating condition without AC control module in function and (2) AC control module operated to keep the temperature within specified range of 19–28 °C for the indoor hydroponic system. Overall system power consumption with and without AC unit is reported in the Table 1. The cost of the total power consumed was calculated using the Tariff calculator from the National utility provider (Kahramaa) of Qatar.

## 4. Results

After the calibration and testing of the sensors and different modules, the complete indoor vertical hydroponic system was implemented and tested. In the initial implementation, it was used to cultivate mint in an indoor area as shown in Figure 3 and the operation of the system was demonstrated in Supplementary Video S1. Later on, lettuce, tomato, cucumber, coriander, capsicum, strawberry, chili seeds, and mint stem were planted in the vertical hydroponic system. In this section, system performance is illustrated using IoT interface in the Thingspeak web-site and mobile application, along with power and water consumption analysis, illustration of plant growth, and comparative analysis between the proposed and other research and commercial projects.

### 4.1. IoT Based Web and Mobile Interface

**Figure 9:**
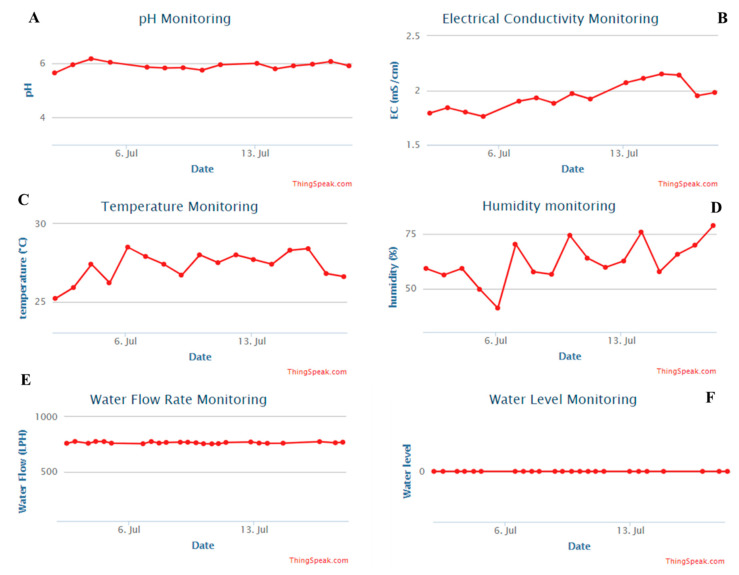
Sample graphs of the Thingspeak Web-interface of IoT platform: (**A**) pH, (**B**) EC, (**C**) Temperature, (**D**) Humidity, (**E**) Water flow rate, and (**F**) Water level monitoring.

**Figure 10:**
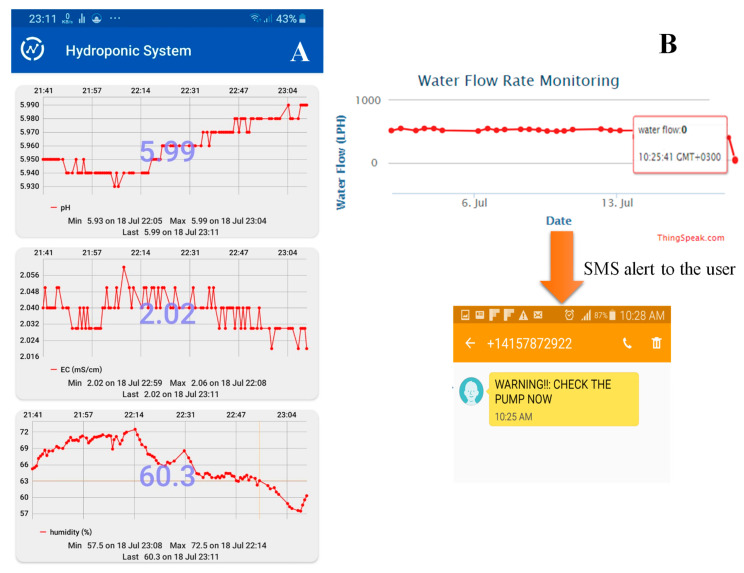
Mobile Application interface for the hydroponic system (**A**) and SMS sent to alert the user when the pump is not working (**B**).

The system was designed to get the data from the sensors and collected in a central microcontroller and send it to IoT platform. IoT platform is capable to store, analyze and preview the data to the user in private or public web-interface and also in a mobile application. The web-interface can be visualized anytime from smart phone or computer and the mobile application is also simple and easily accessible solution. In Figure 9, a sample Thingspeak web-interface is shown. However, Thingspeak web-interface can show several days’ data at a time while the mobile application (Figure 10A) can show for a shorter duration with last data-point highlighted.

As it can be seen from Figure 9 that the whole automated system was successful in maintaining a stable environment favorable for the plants to grow. In Figure 9, it is evident that the system was successful in maintaining the pH and EC within a specified range and whenever it is moving away from the specified range it was controlled by the system as shown in the flowchart of Figure 8. Similarly, the temperature of the area was also maintained in the required level by the automated AC control module to keep the temperature favorable for the plants.

### 4.2. Alerting the User When an Intervention Is Required

An alerting system in the form of text messages has successfully implemented whenever the user intervention is required. The pump is one of the major components that must be continuously on in an automated hydroponic system in order to supply the nutrient solution from the nutrition tank to the system. As a result, any failure in the pump would lead the plants to dry out and die after few hours. Therefore, in case of any failure in the main pump, which is detected by water flow sensor whenever the amount of the water passing through it is 0 L, a short message service (SMS) massage was sent to the user’s phone. It is sent through Thingspeak with an external web (IFTTT web) to inform the user about the failure and to check the pump. Figure 10B shows a message sent immediately to inform the user, when the water flow sensor reading is 0 L.

### 4.3. Power Consumption of the Overall System

One of the major contributions of the paper was the development of a power efficient solution for the continuous monitoring and controlling of the hydroponic system. In order to do that, a continuous power monitoring of the complete system was required. The system was running in two different modes: (1) Artificial light and other modules, and (2) Other modules. The system was consuming 247 watts when artificial light and other modules were working whereas approximately 10 watts was consuming only when the lights were off. Table 1 shows that monthly power consumption from the system is 120.59 kWh when the air conditioner unit was not considered. The cost of the consumed power (per month) is 13.26 QAR. However, the two-ton air conditioning unit that was controlled by the system to maintain the temperature of the room within the specified range was not considered in this cost.

**Figure 11:**
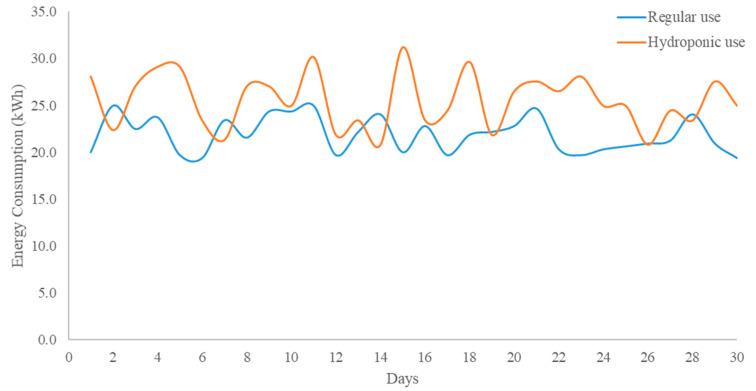
Daily energy consumption due to hydroponic system in comparison to regular use.

Figure 11 shows the variation of daily usage of the air conditioner while in regular use and hydroponic system was controlling the system to keep the temperature within the specified range. It is evident that the overall usage of the AC unit has increased and the monthly consumption from 656 kWh was changed to 766 kWh due to hydroponic system. However, there was a monthly increase of 110 kWh due to hydroponic system based on the study carried out on two consecutive months, where the AC was running in regular use for one month and it was used for hydroponic system for next month. Additional cost due to air conditioned controlled by the hydroponic system is 12.1 QAR. Therefore, the overall monthly system costing including air conditioner’s additional usage is 25.36 QAR.

### 4.4. Water Consumption and Volume Circulation per Month

**Figure 12:**
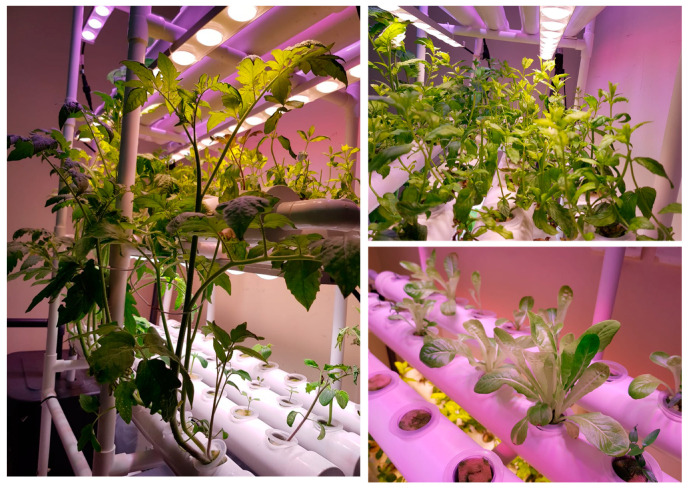
Snapshot of the system in use for growing different plants from seeds and stem in the hydroponic system.

It was recorded that by adding daily circulation of water in the hydroponic system in a month, the system circulates 103.9 k gallon of nutrient solution to the system. Two liters of fresh water was refilled to the system once a week approximately and therefore, monthly water consumption ranges from 8–10 L due to the consumption by the plants and evaporation. Figure 12 shows different plants are growing in the hydroponic system, which is not only producing organic vegetables at the corner of the living room, but has also become a means of enjoyment for the kids as they actively participate in planting the seeds in the system, observing the growth in front of their eyes.

### 4.5. Comparison with Other Similar Research Projects and Commerical Products

Table 2 shows the price and feature comparison of some research projects and commercially available solutions and the proposed solution. It can be seen from the table that the proposed solution offers more features making it independent of human intrusion as much as possible and making it suitable for general people. There are cheaper and incomplete solutions available which do not have IoT solution, very small in size and incapable to produce enough plants and in some cases monitoring or controlling of important parameters are not there. On the other hand, there are very expensive, soilless solutions are available which require huge investment and are not suitable for personal farming. Farmbot is a very nice complete solution however is not suitable for hydroponic farming nor has temperature control feature, which is very important for GCC countries [60].

## 5. Discussion

With the increasing population growth and industrialization, the world is facing food crisis due to the decreasing amount of arable lands. Over a decade, researches have been conducted to find a sustainable solution to increase crop production and reduce water consumption and required land. In 1930, W.E. Gericke first introduced a new method called hydroponics, where plants are cultivated using a nutrient solution without a solid medium for rooting [72,73]. The hydroponic system usage only 10% of the total water resources used in conventional cultivation methods, which can reduce the total water consumption by 5–20 times and land requirements by more than 75% [74,75] for agriculture. Even though cost for installing a hydroponic systems can be ten times higher in comparison to the traditional agriculture production [76], this type of systems allows to control the nutrition required for the plants as roots are in direct contact with the nutrition solution [77]. Many unfavorable aspects of the soil cultivation like plants effected by the soil borne diseases, lack of soil nutrients, and water can be avoided in the hydroponics system [36]. As there are many advantages of the hydroponic system, a cost-effective system is required for large scale cultivation.

There are different techniques of hydroponics systems, such as: wick, drip, ebb-flow, water culture, nutrient film, and aeroponic [78]. After extensive literature review, nutrient film technique (NFT) has been selected for this research [15,36,37,38,44,78]. It is necessary to monitor and control the parameters like humidity, pH, EC, temperature of the surroundings, and water level of the container to ensure the stable and healthy growth of the plants [79,80,81]. In this research, we have implemented an automatic hydroponic system by using internet of things (IoT) platform to realize automatic control to improve the efficiency of planting, precise control of nutrients supplies and environment of the systems. At the same time, it improves the water utilization efficiency and ensures the sound cycle of water resources.

In this automated system, all the sensors were connected to the hydroponic system can be controlled and monitored by the central microcontroller. The relay module was used to control artificial lights, water pump and the dosing pumps that were used to add pH and nutrient to the water. Lastly, all the acquired data from microcontroller circuit were sent wirelessly to an online Thingspeak database through a Wi-Fi module. There is also continuous monitoring of the power consumption using the power meter subsystem which can be expanded for large scale farming. After the implementation of the system, calibration and testing of the sensors and system modules were done and the system was used initially for planting mint from stem in an indoor area and later on, used to plant tomato, strawberry, mint, coriander, cucumber, capsicum, and lettuce. The automated hydroponic system has an alerting system in the form of text messages whenever a user intervention is required. One of the major contributions of the work was the development of continuous power monitoring and an AC controlling module for the hydroponic system. Based on the measured energy consumption by the system, it was calculated that the system was consuming 120.59 kWh without air conditioner and an additional 110 kWh for the air conditioner during peak summer. This is equivalent to the system running cost of 13.26 QAR and 25.36 QAR without and with AC unit cost respectively. Even though the system circulates around 104 k gallons of nutrient in a month, the overall water consumption of the system is only 8–10 L.

Several other studies on the design, implementation, and evaluation of the hydroponic systems were found in the literature with and without IoT and intelligent control mechanism. A comprehensive comparison of these system with the proposed system is discussed in the introduction and also summarizes in Table 2. This automated hydroponic system provides a complete design, including implementation steps, of an indoor solution for monitoring the environmental parameters as well as the control of nutrition and water supply for the healthy and stable growth of plants.

## 6. Conclusions

The core of a hydroponic system is to maintain and control the environmental parameters and the efficient supply of nutrition and water for healthy growth of the plants. In this paper, a cost effective automated vertical hydroponic system using IoT platform has been implemented. The design of the vertical hydroponic system was selected based on a comparison with other designs in terms of costs, efficiency, and suitability to build in small indoor space. The primary structure design of the system has been assembled and the required parameters to build an automatic system were planned in order to select the required components. The parameters of the system were studied and calculated such as the suitable temperature, light wavelength, pH, EC, and the required amount of water for the system. Finally, the parameters were displayed in Thingspeak IoT platform web-interface and mobile application to provide easily accessible user interface. User can monitor visualize the parameters and system can send SMS massage in case of pump interruption. The IoT platform allows to extract data in a CSV file which can help in machine learning algorithm development while the system can produce a large amount data suitable for training classical and deep learning algorithms to enhance the performance of the automated system for controlling. This study has opened up the possibility of carrying out several other potential studies. There is no feasibility study reported in indoor plantation compared to the field vegetation for this region. Moreover, the hydroponically grown plants, organic plants, and field plants can be studied and their comparative growth can be monitored through this wireless platform. In conclusion, this automated cost effective vertical hydroponic system can provide an in-house vegetation solution for the Arab world and its cost will be significantly less if the materials are locally made. Therefore, a wide-spread adoption of such solutions in every household can help to fulfill the local requirements of leafy fresh vegetables and can reduce the dependency on import.

## Figures and Tables

**Table 1:** Power consumption of the individual system component.

Modules	Voltage (V)	Current (A)	Power (W)	Power Consumption (kWh) per Month
pH sensor	5 V DC	$18.3\times 10^{-3}$	0.0915	0.06588
EC sensor	5 V DC	$50\times 10^{-3}$	0.25	0.18
Water pump (Nutrient)	240 V AC	0.033	7.92	5.7
* 2 LED (6K3R4)	240 V AC	2×0.339=0.678	162.72	78.11
* 1 LED (K6)	240 V AC	0.311	74.64	35.83
Water flow sensor	5 V DC	$15\times 10^{-3}$	0.075	0.054
Water level sensor	12 V DC	0.05	0.6	0.432
** Water pump (Fresh water)	12 V DC	0.7	8.4	Negligible
Humidity and temperature sensor	5 V DC	$1.5\times 10^{-3}$	$7.5\times 10^{-3}$	0.0054
Relay	5 V DC	$60\times 10^{-3}$	0.3	0.216
Total monthly power consumption (kWh)				120.59

* Artificial LED lights were kept ON for 16 h in a day. ** This pump runs only for few minutes in couple of days and therefore overall power consumption in a month is negligible.

**Table 2:** Price and feature comparison of some research project and commercial solutions.

Similar Work or Commercial Solution	Small (S) or Large (L) Scale	Soil(S) or Hydroponic(H)	IoT Solution	Light Monitoring (Yes/No)	Temperature Monitoring (M) and Control(C)	pH Monitoring (M) and Control(C)	Price Information	Power Consumption
KOMEOKA ET AL. [66]	L	S	No	Yes	M & C	None	** ~1000$	Not specified
IJAZ ET AL. [67]	L	H	No	Yes	M & C	None	** ~1000$	Not specified
SUGANO ET AL. [68]	L	H	No	Yes	M & C	C	** ~1000$	Not specified
AEROGAR-DEN [69]	S	H	Yes	Yes	None	None	~100$	Not specified
PLANTTALK [28]	S	H but not vertical	Yes	Yes	M & C	M & C	Not specified	Not specified
FARMBOT [70]	S	S	Yes	No	None	C	~1000$	Not available
* PROPOSED SOLUTION	S	H	Yes	Yes	M & C	M & C	~400$	Available

* Not a complete solution, thus the price is an approximate summation of the individual components (i.e., LED lighting systems, Temperature Sensors, Frames and setup) [71]. ** Price includes structure, light and sensors.

## Supplementary Materials

The following are available online at https://www.mdpi.com/1424-8220/20/19/5637/s1, Figure S1: Spectrum of two different LED lights. Figure S2: Illustration of water flow path for the NFT system. Figure S3: pH and EC controlling system’s (A) front view, outlets pipes of dosing pump in the nutrient solution (B), and Nutrient solution, and pH adjustable (Up and Down) are shown connected to the holding box and 3 dosing pumps (C). Figure S4: EC and pH sensor’s performance in different conductivity (up) and pH level (down) measurement respectively. Note: the sensors are very accurate in the working range (EC – 100–10000 µS/cm and pH: 2–10) of the sensors. Table S1: Main Components of the Proposed Architecture. Table S2: GPH required for each system. Table S3: Specifications of hydroponic lights. Table S4: pH and EC range for different plants (considering that the plants have already developed roots.). Video S1: The designed hydroponic system.
