# Supplementary material for: Design, Construction and Testing of IoT Based Automated Indoor Vertical Hydroponics Farming Test-Bed in Qatar
Source: Sensors (Basel). 2020 Oct 2;20(19):5637. doi: 10.3390/s20195637 (PMC7582991; doi:10.3390/s20195637)
Supplement: Supplementary file 1 [file sensors-20-05637-s001.pdf]

Table S1. Main components of the proposed architecture.

**Artificial Lights** 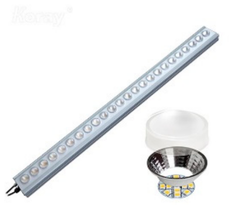	Three LED lights from Koray Company for three tiers of hydroponic system were used. Two lights were type 6K3R4, and the other one was type K6. The light spectrum test shows that 6K3R4 has high intensity of the red light which makes it suitable for growing leafy vegetables and for the K6, it has high intensity of blue and red lights which will make it suitable for growing flowering plants.
**Arduino Mega 2560** 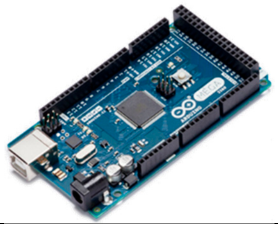	All the sensors were connected to this microcontroller and the sensor data were transmitted to the cloud using the Wi-Fi module from this microcontroller.
**Arduino Nano** 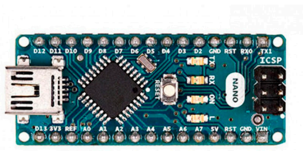	The power measurement module were kept isolated for safety and constructed around an Arduino Nano Microcontroller which sent power measurements to the central Arduino Mega Microcontroller.
**Wi-Fi module ESP8266** 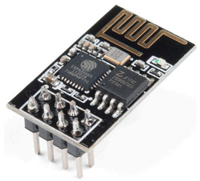	Sensor data were transmitted to the IoT platform using this module.
**Submersible Water Pump (inside the nutrient container)** 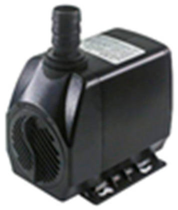	This water pump was used to pump the nutrient solution continuously to the hydroponic system, which can supply water to higher head height and the flow volume of the pump can be controlled manually by the regulator of the pump.
**Submersible Water Pump (inside the water container)** 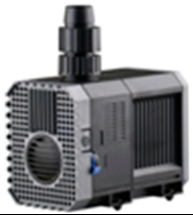	A second pump was placed inside the fresh water container which is used as water source to supply water to the nutrient container. It supplied additional fresh water whenever the water level sensor detects a drop in the level of the water.
**Water Flow Sensor** 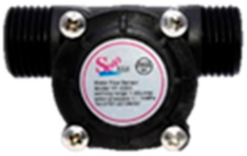	A YF-S201 Hall-Effect flow sensor was placed between the water pump and the hydroponic system. It was used to detect if the pump was pumping sufficient amount of nutrient solution into the hydroponic system. If the pump is not working, the user will be notified via a text message as shown in Figure 9.

**Water Level Sensor**<br>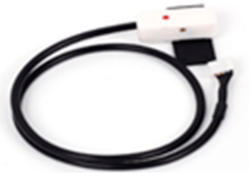	A non-contact capacitive water level sensor was attached outside the container, if the level of nutrient solution inside the container goes below the pre-specified level of 22.5cm (which is equivalent to 37L), the water level sensor triggers the fresh water pump on to refill the required amount of water in the nutrient container.
**pH Sensor**<br>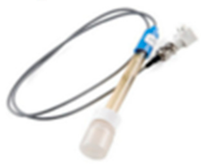	Atlas Scientific pH Kit 0-14 pH sensor was used to real-time measure the pH value in the nutrient container.
**EC Sensor**<br>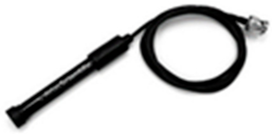	Atlas Scientific Conductivity Kit K 1.0 5-200,000 μS/cm sensor is used to real-time measure the nutrition concentration in the nutrient container.
**Dosing Pumps**<br>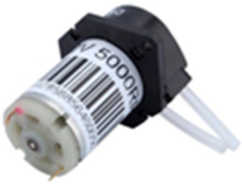	Three dosing pumps were used in the system where two dosing pumps were used to adjust the pH level. If the pH sensor detects a drop or an increase in the pH level in the nutrient solution, the corresponding dosing pump will run to add (pH up) or (pH down) solution to adjust the pH and the third dosing pump was used to add the nutrient solution to the nutrient container.
**Dosing Pumps Holder**<br>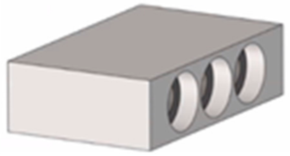	A custom design box that is designed in order to hold all the dosing pumps.
**Temperature and Humidity Sensor**<br>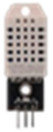	DHT22 sensor was used to measure temperature and humidity of the hydroponic area.
**16 channel Relay**<br>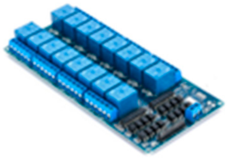	Artificial lights, dosing pumps, and water supply pump are connected to the relay to switch them ON and OFF depending on the requirements of the system.
**Real Time Clock**<br>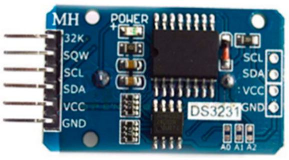	DS3231 RTC module was interfaced to the central MCU to maintain real time clock running in the system so that timely event can be maintained properly.

Table S2. GPH required for each system.

Hydroponics		
**Type**	**Flow Rate GPH**	**Total GPH**
Zip Grow Towers	2 GPH per Tower	= (# of towers) (Flow rate GPH)
Deep water culture	Tank volume in gallons/hr	= (# of volume) (Flow rate GPH)
NFT	4-6 GPH per trough	= (# of troughs) (Flow rate GPH)

Table S3. Specifications of hydroponic lights.

Model Type	Dominated Color in the Spectrum	Peak Wavelength	Electrical Parameters
1. 6K3R4	Red	660 nm	V = 24 V, I = 0.7582 A, P = 18.20 W
2. K6	Red and blue	645 nm	V = 231.5 V, I = 0.08053 A, P = 17.9 W

Table S4. pH and EC range for different plants (considering that the plants have already developed roots.).

Plant	EC (mS/cm)	pH
**Lettuce**	0.56–0.84	6.0–6.5
**Mint**	1.4–1.68	5.5–6.0
**Celery**	1.26–1.68	6.0–6.5
**Cabbage**	1.75–2.10	6.5–7.0
**Spinach**	1.260–1.61	6.0–7.0
**Kale**	0.56–0.84	6.0–6.5
**Beans**	1.40–2.80	5.5–6.5
**Strawberry**	1.26–1.68	6.0

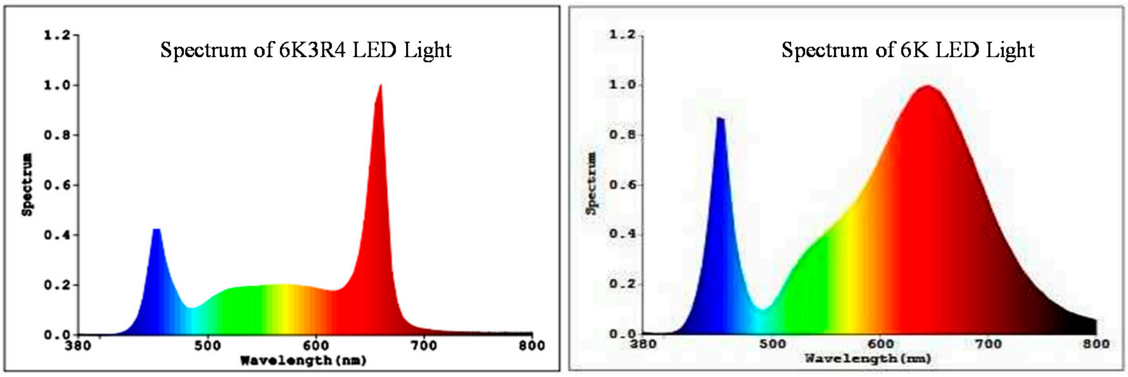

Figure S1. Spectrum of two different LED lights.

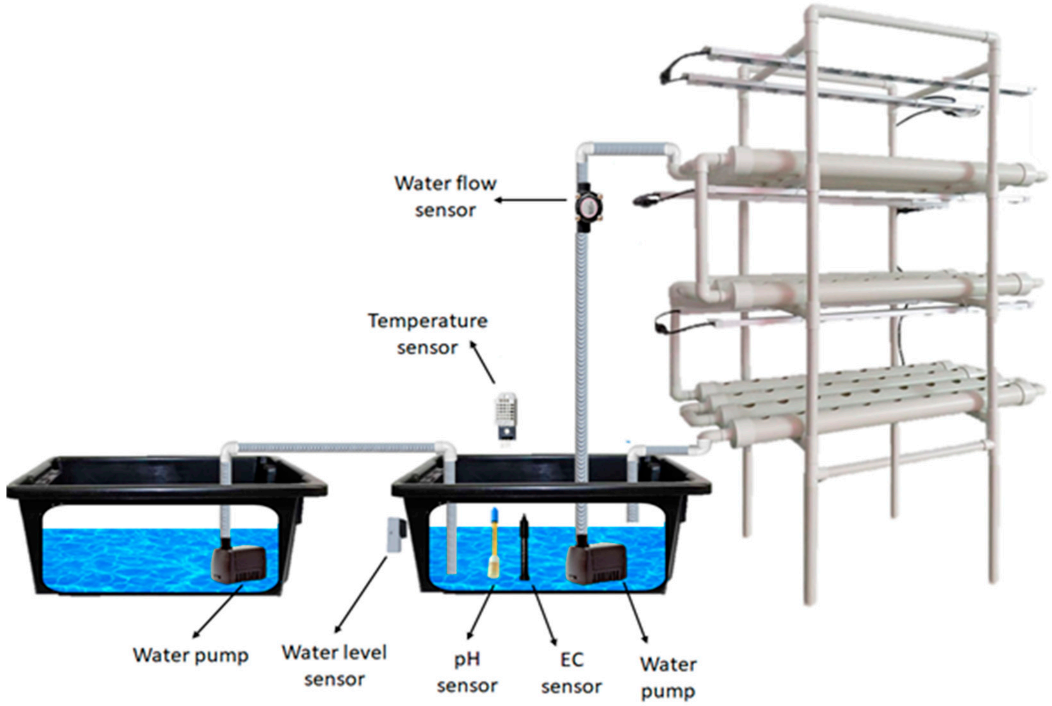

Figure S2. Illustration of water flow path for the NFT system.

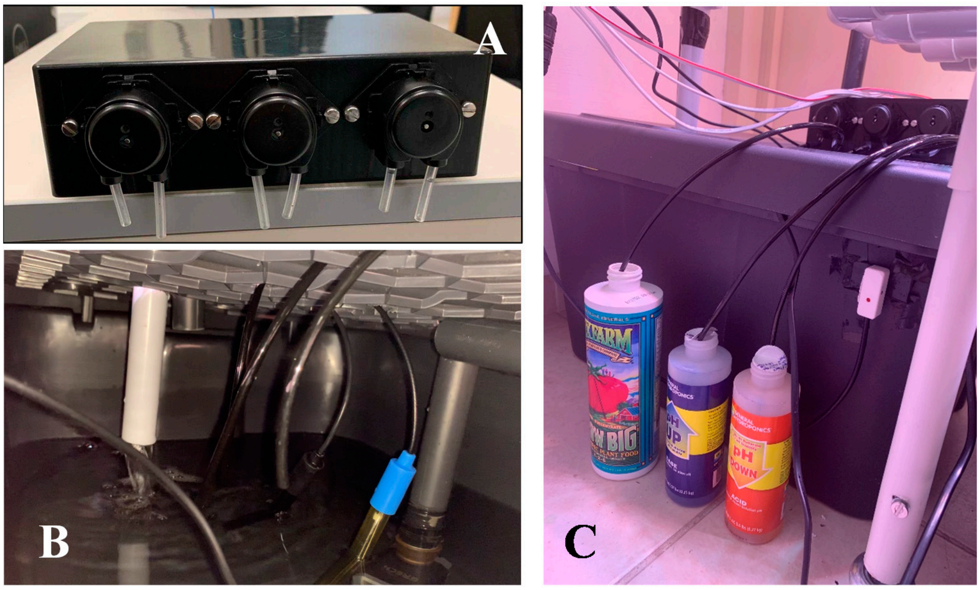

Figure S3. pH and EC controlling system's (**A**) front view, outlets pipes of dosing pump in the nutrient solution (**B**), and Nutrient solution, and pH adjustable (Up and Down) are shown connected to the holding box and 3 dosing pumps (**C**).

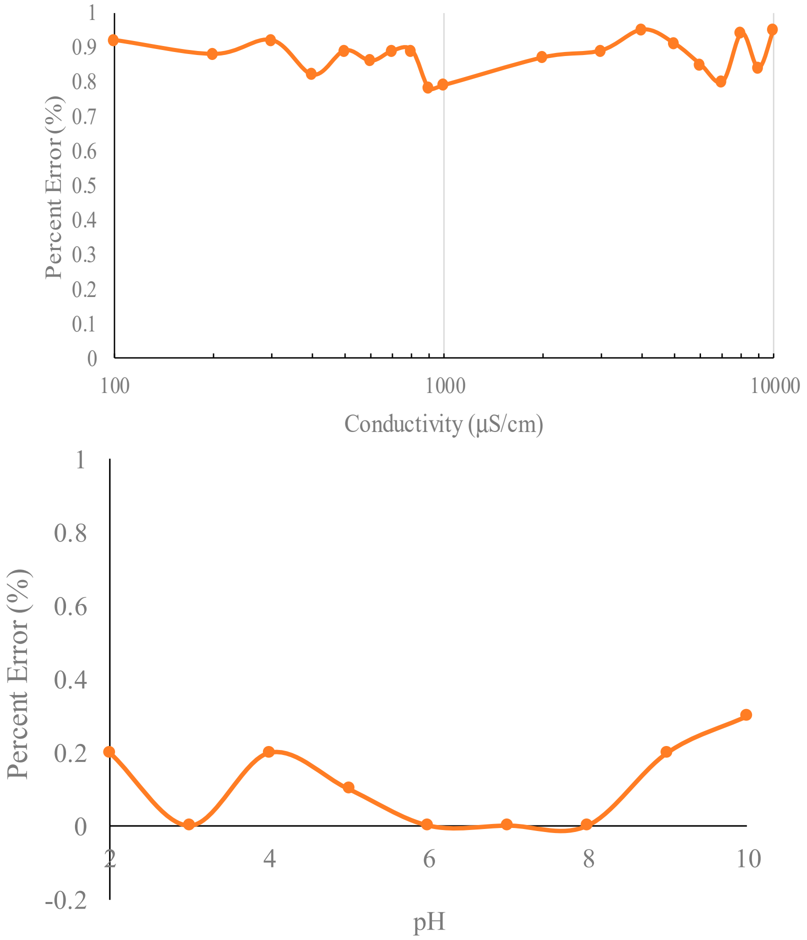

Figure S4. EC and pH sensor's performance in different conductivity (up) and pH level (down) measurement respectively. Note: the sensors are very accurate in the working range (EC- 100-10000 µS/cm and pH: 2-10) of the sensors.

https://drive.google.com/file/d/1qVCFErQLQIG4Z9Vj3Fjoq4SVx4QUs0_q/view?usp=sharing

Video S1. The designed hydroponic system.
